# Progranulin-derived granulin E and lysosome membrane protein CD68 interact to reciprocally regulate their protein homeostasis

**DOI:** 10.1016/j.jbc.2022.102348

**Published:** 2022-08-04

**Authors:** Mariela Nunez Santos, Daniel H. Paushter, Tingting Zhang, Xiaochun Wu, Tuancheng Feng, Jiaoying Lou, Huan Du, Stephanie M. Becker, Robert Fragoza, Haiyuan Yu, Fenghua Hu

**Affiliations:** 1 Department of Molecular Biology and Genetics, Weill Institute for Cell and Molecular Biology, Cornell University, Ithaca, New York, USA; 2 Department of Gynecology, Dongfang Hospital, Beijing University of Chinese Medicine, Beijing, P. R. China; 3 Department of Computational Biology, Weill Institute for Cell and Molecular Biology, Cornell University, Ithaca, New York, USA

**Keywords:** progranulin, granulin, CD68, lysosome, frontotemporal dementia, microglia, AP, alkaline phosphatase, BCA, bicinchoninic acid, BMDM, bone marrow–derived macrophage, cDNA, complementary DNA, co-IP, coimmunoprecipitation, DMEM, Dulbecco’s modified Eagle’s medium, GCase, glucocerebrosidase, GRN, granulin, HBH, Hank’s balanced salt solution with Hepes buffer, LAMP, lysosomal-associated membrane protein, MW, molecular weight, PGRN, progranulin, PSAP, prosaposin, TM, transmembrane

## Abstract

Progranulin (PGRN) is a glycoprotein implicated in several neurodegenerative diseases. It is highly expressed in microglia and macrophages and can be secreted or delivered to the lysosome compartment. PGRN comprises 7.5 granulin repeats and is processed into individual granulin peptides within the lysosome, but the functions of these peptides are largely unknown. Here, we identify CD68, a lysosome membrane protein mainly expressed in hematopoietic cells, as a binding partner of PGRN and PGRN-derived granulin E. Deletion analysis of CD68 showed that this interaction is mediated by the mucin–proline-rich domain of CD68. While CD68 deficiency does not affect the lysosomal localization of PGRN, it results in a specific decrease in the levels of granulin E but no other granulin peptides. On the other hand, the deficiency of PGRN, and its derivative granulin peptides, leads to a significant shift in the molecular weight of CD68, without altering CD68 localization within the cell. Our results support that granulin E and CD68 reciprocally regulate each other’s protein homeostasis.

Frontotemporal lobar degeneration is a progressive neurodegenerative disease characterized by changes in personality and behavior as well as cognitive decline and language impairments (https://www.uptodate.com/contents/frontotemporal-dementia-epidemiology-pathology-and-pathogenesis) (1, 2). Mutation in the *granulin* (*GRN*) gene, resulting in haploinsufficiency of the progranulin (PGRN) protein, is one of the leading causes of frontotemporal lobar degeneration (3, 4, 5), with over 70 disease-associated GRN mutations identified (https://www.uptodate.com/contents/frontotemporal-dementia-epidemiology-pathology-and-pathogenesis). PGRN is a secreted glycoprotein that is involved in many cellular processes, including inflammation, wound healing, and tumorigenesis (6, 7). PGRN is widely considered to be anti-inflammatory, aiding in the reduction of proinflammatory cytokines and reducing the activation of microglia and astrocytes (7, 8, 9). Structurally, PGRN is comprised of 7.5 granulin segments, denoted as granulin A, B, C, D, E, F, and G, and the half-granulin segment known as paragranulin (6, 7). The granulin peptides have been shown to possess functions that are independent of PGRN (6, 7, 8).

In addition, accumulating evidence supports that PGRN is critical for proper lysosomal function (8, 10). Complete loss of PGRN, caused by homozygous mutations in the *GRN* gene, leads to neuronal ceroid lipofuscinosis, a lysosomal storage disorder that is characterized by degeneration of nerve cells and the accumulation of autofluorescent lipofuscin (11, 12). PGRN is a resident lysosomal protein and trafficked to the lysosome *via* two independent pathways, directly by the sortilin receptor (13) or indirectly by binding to the soluble lysosomal protein, prosaposin (PSAP), which carries PGRN with it to the lysosome when it binds its own trafficking receptors, low-density lipoprotein receptor–related protein 1 or the cation-independent mannose-6-phosphate receptor (14). Within the lysosome, PGRN is processed into individual granulin peptides by lysosomal proteases (15, 16, 17, 18), and these granulin peptides have been proposed to be the functional units of PGRN to regulate lysosomal functions. In support of this, the activities of several lysosomal enzymes, including cathepsin D (19, 20, 21, 22), glucocerebrosidase (GCase) (23, 24, 25, 26), and asparagine endopeptidase (18), have been shown to be affected by the loss of PGRN and granulin peptides. PGRN deficiency results in the accumulation of myelin debris in microglial lysosomes, which is further exacerbated by the reduction in cathepsin D levels (27). In addition, PGRN was shown to interact with bis(monoacylglycero)phosphate, an endolysosomal phospholipid, and PGRN loss leads to a significant reduction in bis(monoacylglycero)phosphate levels (26). However, the function of each granulin peptide remains to be characterized. Interestingly, the levels of individual granulin peptides are differentially regulated despite being derived from the same precursor (28), but the mechanisms involved in modulating the stability of individual granulins in the lysosome remain unknown.

Here, we report the identification of CD68 as a binding partner for full-length PGRN and granulin E. CD68 is a type I transmembrane (TM) protein that is abundantly expressed in microglia and macrophages and localized to the plasma membrane, late endosomal membrane, and lysosomal membrane (29). Although the exact function of CD68 remains to be determined, our data support that CD68 and granulin E regulate each other’s homeostasis in the lysosome.

## Results

### PGRN and granulin E interact with CD68

**Figure 1:**
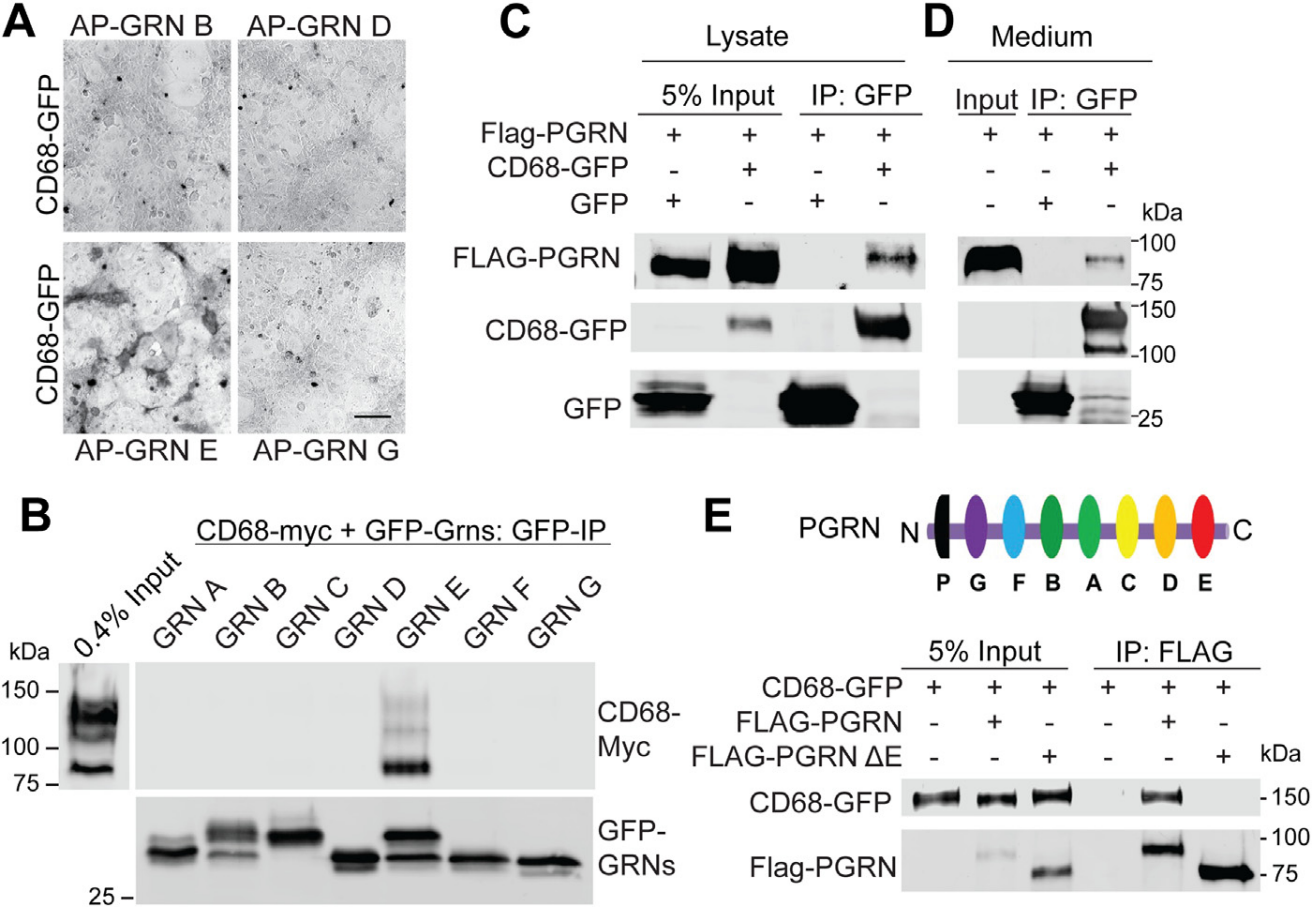
**Physical interaction between CD68 and granulin E.***A*, COS7 cells transfected with CD68-GFP were incubated with conditioned media containing AP-tagged granulin peptides. Positive interaction between CD68 and the granulin E was observed after incubation with AP substrate. The scale bar represents 100 μm. *B*, Myc-His-tagged CD68 and GFP-tagged granulin A through G were transfected in HEK293T. Lysates were incubated with anti-GFP-conjugated beads. After washes, products were analyzed by Western blot using anti-GFP and anti-Myc antibodies as indicated. *C*, FLAG-tagged PGRN and GFP-tagged CD68 were transfected in HEK293T cells. Lysates were incubated with anti-GFP-conjugated beads. After washes, products were analyzed by Western blot using anti-GFP and anti-FLAG antibodies as indicated. *D*, conditioned media containing FLAG-tagged PGRN were incubated with beads bound to GFP or GFP-CD68. After washes, products were analyzed by Western blot using anti-GFP and anti-FLAG antibodies as indicated. *E*, HEK293T cells were transfected with GFP-tagged CD68 and FLAG-tagged PGRN or PGRN-ΔE. Lysates were incubated with anti-FLAG-conjugated beads. After washes, products were analyzed by Western blot using anti-GFP and anti-FLAG antibodies as indicated. AP, alkaline phosphatase; HEK293T, human embryonic kidney 293T cell line; PGRN, progranulin.

To identify potential TM protein-binding partners of PGRN or granulin peptides, we cherry-picked 2815 complementary DNAs (cDNAs) encoding the TM proteins from the human ORFeome 8.1 library. The cDNAs were cloned into a mammalian vector containing a C-terminal GFP tag and transfected into COS-7 cells. The cells were incubated with alkaline phosphatase (AP)–tagged PGRN or granulin peptides. Positive interactions were visualized using AP substrates following a previously published protocol (30). From this screen, we identified the TM protein, CD68, as a binding partner for granulin E (Fig. 1*A*). To further confirm these interactions, we performed coimmunoprecipitation (co-IP) assays of CD68 with full-length PGRN or granulin peptides and found that CD68 binds to both full-length PGRN and granulin E but no other granulins (Fig. 1, *B*–*D*). Deletion of the C-terminal fragment of PGRN, which contains the granulin E domain (PGRNΔE), abolishes the interaction with CD68 (Fig. 1*E*), further supporting that CD68 binds to PGRN *via* the granulin E domain.

**Figure 2:**
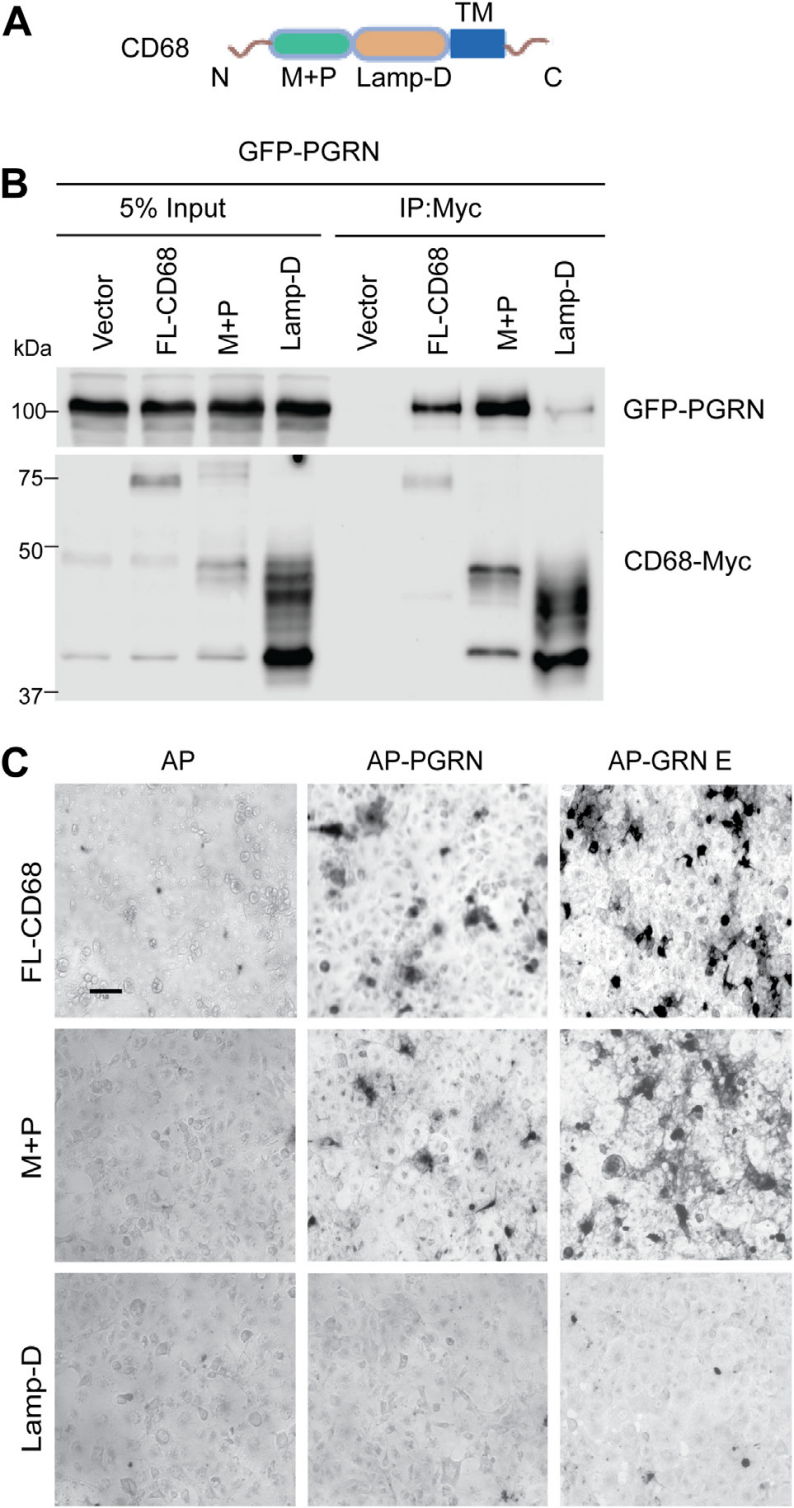
**CD68 binds PGRN and granulin E *via* the mucin–proline-rich domain.***A*, schematic of CD68 domain structure: mucin–praline-rich domain (M + P) or the LAMP-like domain (Lamp-D), and the transmembrane (TM) domain. *B*, Myc-tagged CD68 constructs containing the M + P or Lamp-D domains were expressed in HEK293T cells with GFP-tagged PGRN, and the lysates were coimmunoprecipitated using anti-Myc-conjugated beads. IP products were analyzed by Western blot and probed with Myc and GFP antibodies as indicated. Full length-CD68 (FL-CD68) is used as a control. *C*, M + P or the Lamp-D domains of CD68 were fused to the transmembrane domain of PDGFR (pDisplay vector; Invitrogen) and transfected into COS-7 cells. FL-CD68 is used as a control. Cells were incubated with AP-tagged PGRN and granulin E (100 nM). The scale bar represents 100 μm. AP, alkaline phosphatase; HEK293T, human embryonic kidney 293T cell line; IP, immunoprecipitation; LAMP, lysosomal-associated membrane protein; PDGFR, platelet-derived growth factor receptor; PGRN, progranulin.

CD68 contains three domains: the TM domain, the mucin–proline-rich region (M+P), and the lysosomal-associated membrane protein (LAMP)-like domain (Lamp-D) (29). To characterize which regions of CD68 mediate the interaction with granulin E, we generated deletion constructs of CD68. Deletion of the mucin–proline-rich domain, but not the LAMP-like domain, abolished CD68 binding to PGRN and granulin E (Fig. 2, *A,* *B* and C), supporting that the mucin–proline-rich domain of CD68 is required for the interaction with PGRN and granulin E.

### CD68 is not an essential PGRN trafficking receptor

**Figure 3:**
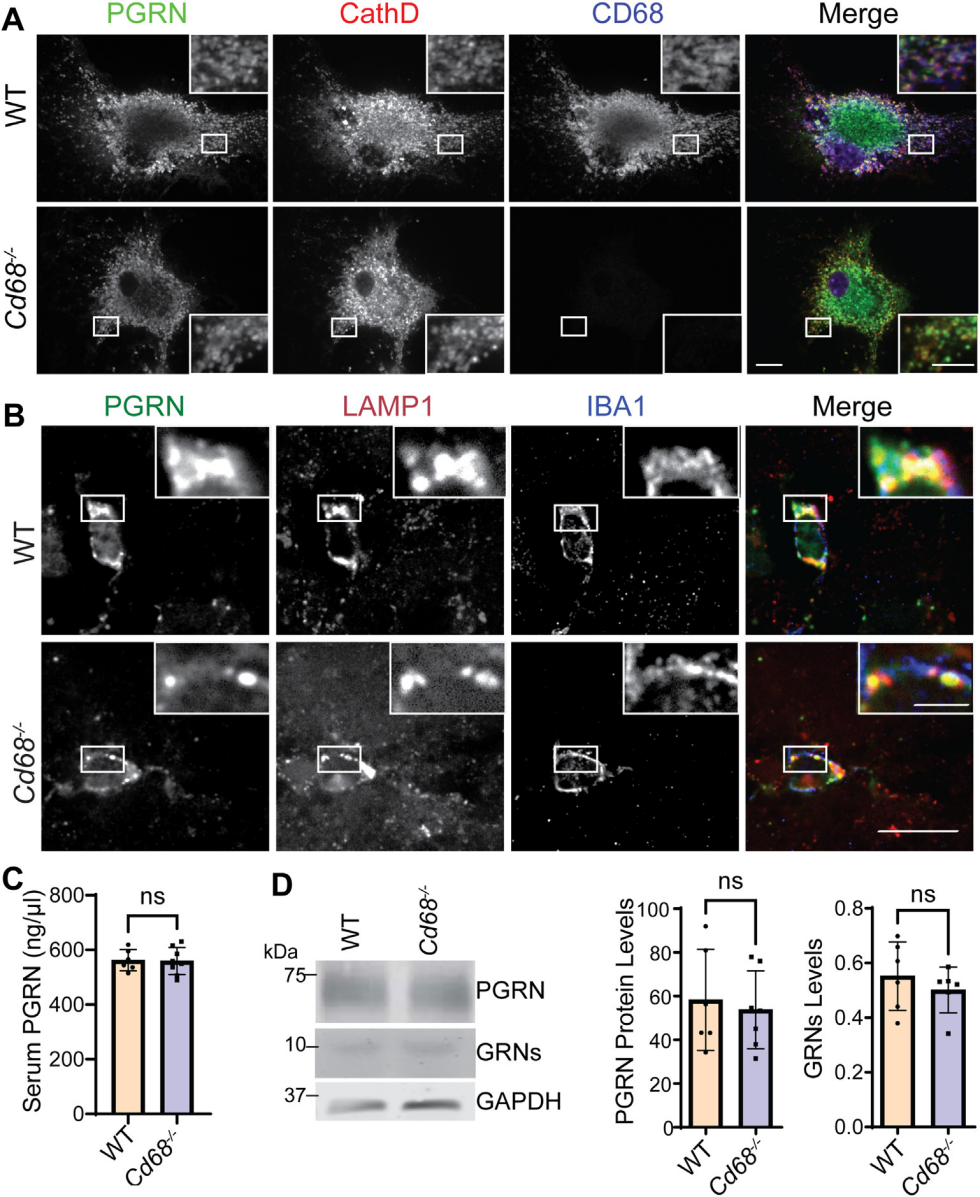
**CD68 is not required for PGRN lysosomal trafficking.***A*, WT and *Cd68*^*−/−*^ bone marrow–derived macrophages (BMDMs) were stained with anti-cathepsin D (CathD), PGRN, and CD68 antibodies. The scale bar represents 10 μm (*inset*: 5 μm). *B*, brain sections from adult WT and *Cd68*^*−/−*^ mice were stained with anti-PGRN, LAMP1, and IBA1 antibodies. The scale bar represents 100 μm (*inset*: 25 μm). Representative images from three mice of each genotype were shown. *C*, serum PGRN levels in WT and *Cd68*^*−/−*^ mice were measured by ELISA. Six mice per genotype were analyzed (n = 6). *D*, Western blot analysis of PGRN and granulin peptides in spleen lysates of WT and *Cd68*^*−/−*^ mice. Six mice per genotype were analyzed (n = 6). PGRN and granulin intensities were normalized to GAPDH. LAMP1, lysosomal-associated membrane protein 1; ns, not significant; PGRN, progranulin.

**Fig S1:**
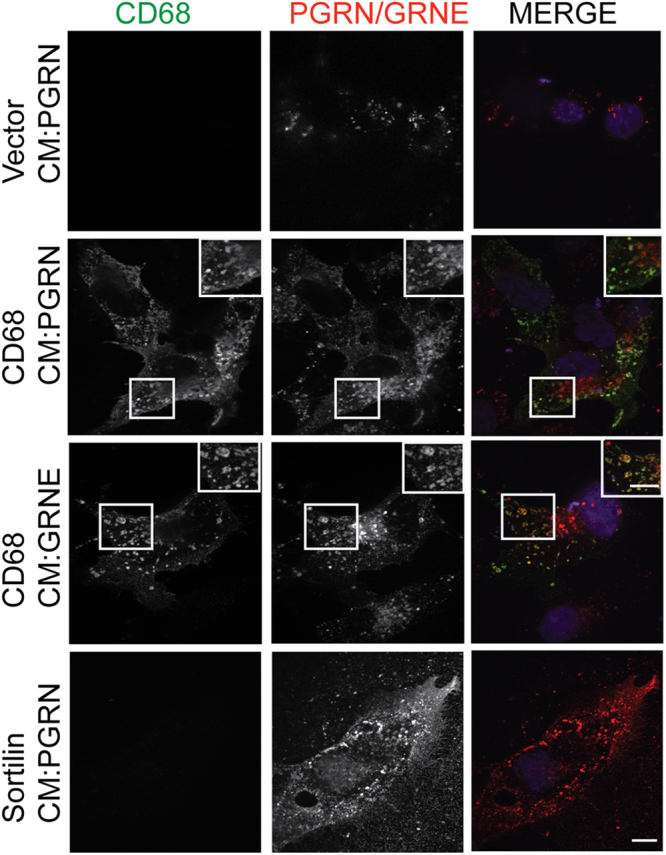
**CD68 can mediate the uptake of PGRN and granulin E when overexpressed.** COS-7 cells were transfected with vector control, CD68 or sortilin-expressing constructs and incubated with conditioned medium (CM) containing PGRN or granulin E as indicated. The cells were washed and fixed 4 hours after incubation and stained with CD68 and PGRN antibodies. Scale bar = 10 μm.

Since CD68 is present in both the plasma membrane and the lysosomal membrane, we hypothesized that CD68 might function as a PGRN lysosomal trafficking receptor. To test this, we transfected CD68 into COS-7 cells and incubated the cells with the conditioned medium containing PGRN. Sortilin-transfected cells were used as a positive control. Like sortilin, CD68 is able to mediate the uptake of PGRN but much less robustly (Fig. S1). Next, we examined the lysosomal localization of PGRN in *Cd68*^*−/−*^ cells. We found that PGRN remains colocalized with lysosomal markers cathepsin D and LAMP1 of cultured *Cd68*^*−/−*^ macrophages (Fig. 3*A*) and *Cd68*^*−/−*^ microglia in the mouse brain (Fig. 3*B*). Thus, although CD68 can potentially mediate PGRN uptake, ablation of CD68 does not have any obvious effect on PGRN lysosomal trafficking *in vivo*.

Defects in PGRN lysosomal trafficking often lead to increased levels of PGRN in the extracellular space, such as in the case of sortilin or PSAP deficiency (13, 14). If CD68 were a PGRN lysosomal trafficking receptor, we may expect that CD68 deficiency would impair PGRN lysosomal trafficking and thus, cause an increase in the extracellular levels of PGRN. However, the levels of PGRN in the serum were not altered in *Cd68*^*−/−*^ mice (Fig. 3*C*). PGRN is processed to granulin peptides in the lysosome, and defects in PGRN trafficking lead to PGRN processing defects (15). To determine whether CD68 affects PGRN processing, we determined the levels of granulin peptides in the spleen, where CD68 is highly expressed. The overall levels of full-length PGRN and granulin peptides are not altered in CD68-deficient spleen lysates (Fig. 3*D*), further supporting that CD68 is not an essential PGRN lysosomal trafficking receptor.

### CD68 deficiency decreases the levels of granulin E

**Figure 4:**
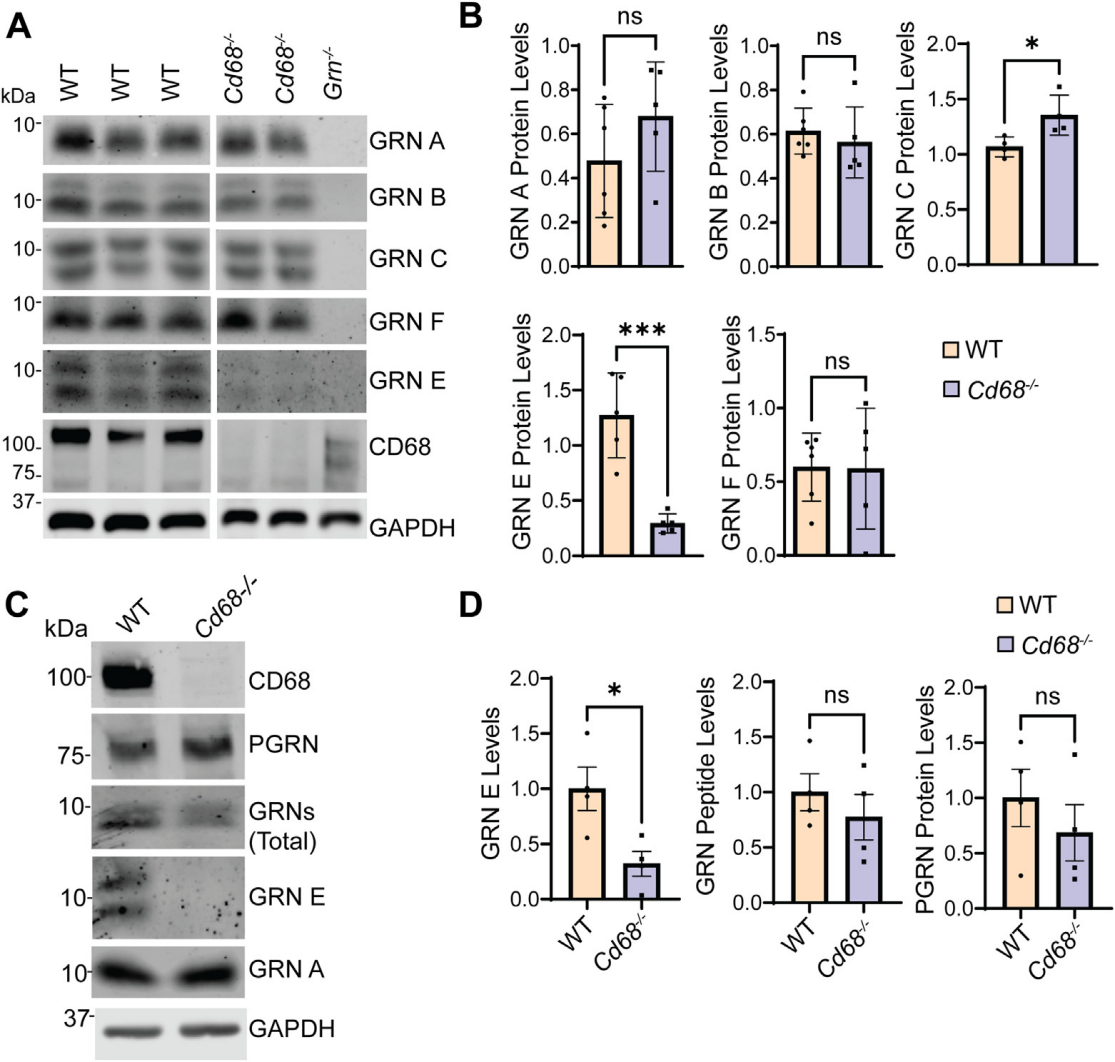
**CD68 deficiency causes a specific reduction in the levels of granulin E.***A* and *B*, spleen lysates of 3-month-old WT and *Cd68*^*−/−*^ mice were analyzed *via* Western blot and probed with antibodies against individual granulin peptides as indicated. The levels of granulin peptides A, B, C, E, and F were quantified and normalized to GAPDH. Six mice per genotype were analyzed (n = 6). ns, not significant; ∗*p* < 0.05, ∗∗∗*p* < 0.001, unpaired *t-*test. *C* and *D*, lysates from WT and *Cd68*^*−/−*^ microglia were probed with antibodies against individual granulin peptides as indicated. The levels of granulin peptides A and E relative to GAPDH were quantified and normalized to WT. Results were from three sets of microglia cultured independently from mouse pups of corresponding genotypes (n = 3). ∗*p* < 0.05, ns, not significant; unpaired *t*-test.

Since CD68 specifically interacts with granulin E and the granulin E domain of PGRN, next we tested whether CD68 deficiency alters granulin E levels. To do this, we generated homemade antibodies against individual granulin peptides (28) and measured the levels of granulin E and other granulins in the lysate prepared from the spleen tissue and primary microglia, which have high levels of CD68 expression. Antibodies against granulin A, B, C, E, and F, but not D and G, can detect endogenous granulin peptides specifically (Fig. 4*A*) (28). Notably, our results show that there is a significant reduction in the levels of granulin E, but no other granulins in *Cd68*^*−/−*^ spleen lysates (Fig. 4, *A* and *B*) and primary microglia cultured from *Cd68*^*−/−*^ pups (Fig. 4, *C* and *D*).

The specific reduction of granulin E in *Cd68*^*−/−*^ samples could be due to altered PGRN processing, reduced stability of granulin E in the lysosome, or changes in granulin E secretion or endocytosis. To determine whether CD68 regulates granulin E trafficking, we incubated CD68-expressing COS-7 cells with conditioned medium containing granulin E. We found that CD68 is able to mediate the endocytosis and lysosomal delivery of granulin E when overexpressed in COS-7 cells (Fig. S1), indicating that granulin E trafficking could be affected by CD68. However, we failed to detect a clear signal for granulin E in the media of *Cd68*^*−/−*^ primary microglia, even after trichloroacetic acid precipitation or immunoprecipitation (data not shown), indicating that the majority of granulin E is intracellular and the decrease in granulin E levels in CD68 deficient cells is unlikely because of increased secretion or decreased endocytosis.

### CD68 deficiency does not affect cathepsin D or GCase activities or PSAP processing

**Fig S2:**
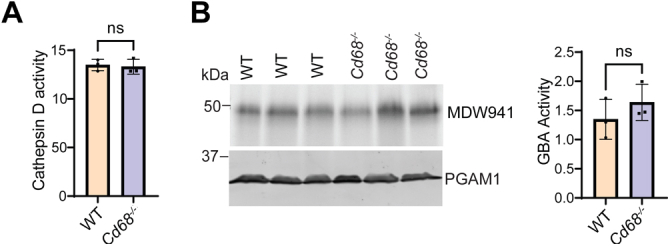
**CD68 deficiency does not affect cathepsin D or GCase activities.***A,* spleen lysates from 6.5-month-old WT and *Cd68*^−^*^/^*^−^ mice were incubated with fluorogenic cathepsin D substrate. Fluorescence was read at 340 nm. Three mice per genotype were analyzed (n = 3). ns, not significant. *B,* spleen lysates of 10-month-old WT and *Cd68*^−^*^/^*^−^ mice were incubated with the GCase activity probe, MDW941. The samples were run on SDS-PAGE, and MDW941-labeled GCase was detected using a fluorescent scanner at 532-nm excitation. Western blot was performed using anti-PGAM1 antibodies as a loading control. Three mice per genotype were analyzed (n = 3). ns, not significant.

**Fig S3:**
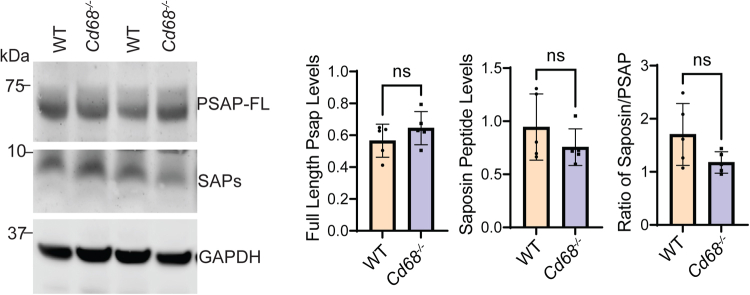
**CD68 deficiency does not affect Prosaposin processing.** Spleen lysates from 10-month-old WT and *Cd68*^−^*^/^*^−^ mice were analyzed via Western blot and probed with anti-prosaposin (PSAP) antibodies. The levels of PSAP and saposin peptides were quantified and normalized to GAPDH. Five mice per genotype were analyzed (n = 5). ns, not significant; unpaired *t*-test.

Since CD68 deficiency leads to reduced levels of granulin E and granulin E has been shown to regulate GCase (31) and cathepsin D activities (19, 21), we determined whether loss of CD68 would affect GCase and cathepsin D activities. No obvious difference was found between WT and *Cd68*^*−/−*^ spleen lysates (Fig. S2, *A* and *B*). In addition, we analyzed whether the levels of PSAP and saposins are altered by CD68 loss since PSAP interacts and traffics with PGRN to the lysosome (32), and among all the granulins, granulins D and E have a strong interaction with PSAP (33). The levels of both full-length PSAP and saposins are not altered by CD68 ablation (Fig. S3), indicating that CD68 does not affect PSAP expression or processing.

### PGRN deficiency affects CD68 homeostasis

**Figure 5:**
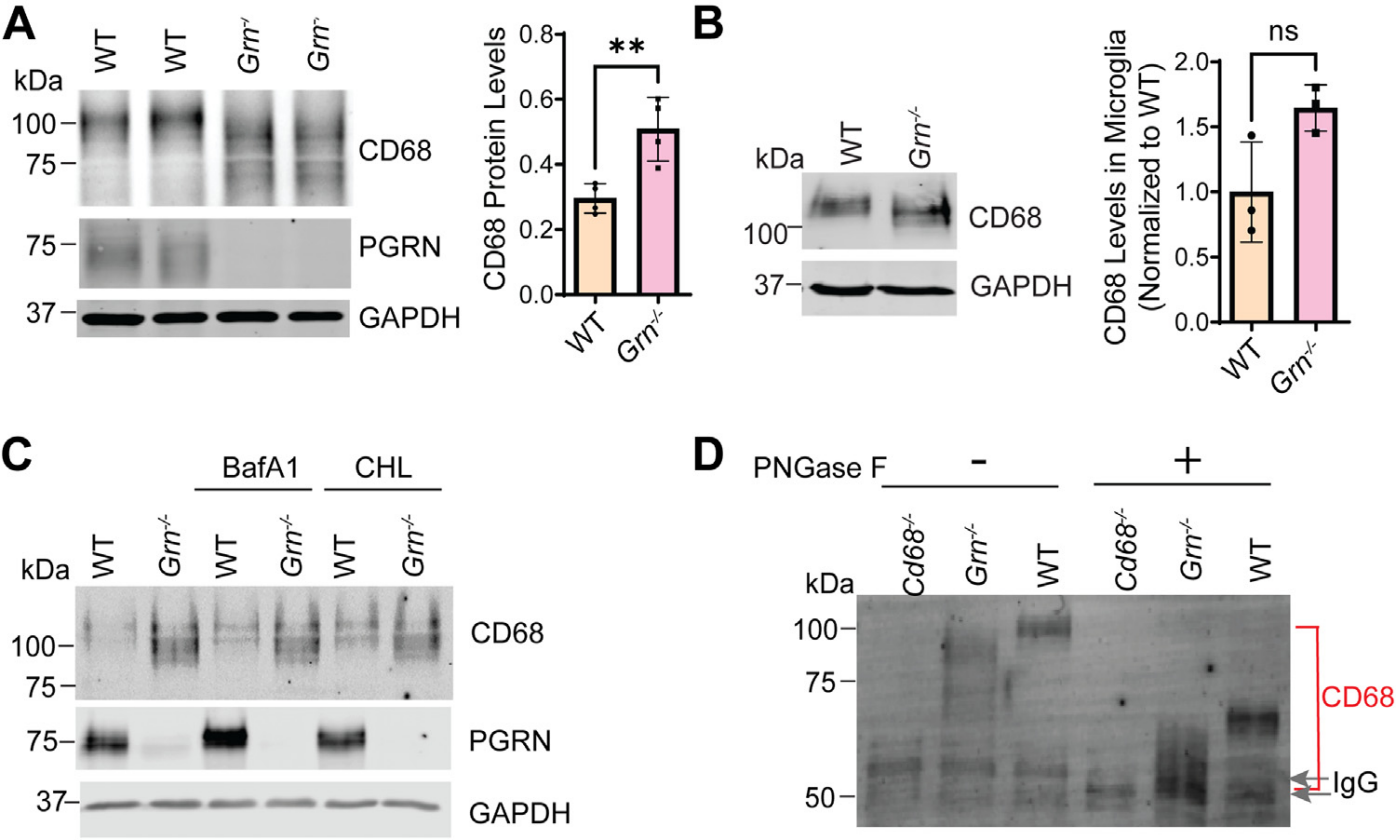
**PGRN deficiency leads to decreased molecular weight of CD68.***A*, Western blot analysis of CD68 in WT and *Grn*^*−/−*^ spleen lysates. CD68 levels were quantified and normalized to GAPDH. n = 4, ∗∗*p* < 0.01, unpaired *t-*test. *B*, WT and *Grn*^*−/−*^ BMDM cells were treated with lysosomal inhibitors, bafilomycin (50 nM), and chloroquine (250 μM) for 8 h at 37 °C. Representative blots from two replicates were shown. *C*, Western blot analysis of CD68 in primary microglia derived from WT and *Grn*^*−/−*^ mice. Results were from three sets of microglia cultured independently from mouse pups of corresponding genotypes (n = 3). ns, not significant; unpaired *t-*test. *D*, spleen lysates from WT, *Grn*^*−/−*^, and *Cd68*^*−/−*^ mice were immunoprecipitated using rat antimouse CD68 antibodies. The beads were then incubated with or without PNGase F. Representative blots from three replicates are shown. Immunoglobulin G (IgG) bands are indicated by *arrows*. BMDM, bone marrow–derived macrophage; PGRN, progranulin.

To explore how the deficiency of PGRN and granulin peptides affect CD68 protein homeostasis, we examined CD68 protein in *Grn*^*−/−*^ cells and tissues. Western blot analysis of *Grn*^*−/−*^ spleen lysates showed a decrease in the molecular weight (MW) of CD68 in addition to an increase in CD68 levels (Fig. 5*A*). MW changes of CD68 are also seen in PGRN-deficient primary microglia and bone marrow–derived macrophages (BMDMs) (Fig. 5, *B* and *C*). Since CD68 is a resident lysosomal glycoprotein, the MW shift could be indicative of changes in lysosomal cleavage or glycosylation of CD68. To test the former, we treated the cells with bafilomycin and chloroquine, which are agents that alter the lysosomal pH and thus decrease lysosomal degradative capacity. CD68 MW in *Grn*^*−/−*^ cells is not altered by lysosome inhibition (Fig. 5*C*), suggesting that CD68 MW changes under PGRN-deficient conditions are not because of lysosome-dependent cleavage.

To determine whether alterations in N-linked glycosylation are responsible for the changes in CD68 MW, PNGase F was used to remove N-linked glycosyl groups from CD68 immunoprecipitated from either WT or *Grn*^*−/−*^ spleen lysates. The MW shift of CD68 is still present in *Grn*^*−/−*^ samples upon the removal of all N-glycans (Fig. 5*D*), indicating that the MW change of CD68 in PGRN-deficient conditions is not due to alterations in N-glycosylation.

**Fig S4:**
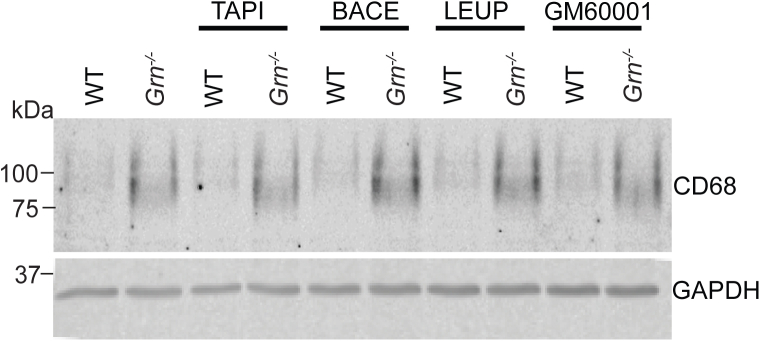
**Inhibition of proteases does not rescue CD68 molecular weight changes in PGRN**-**deficient cells.** WT and *Grn*^−^*^/^*^−^ BMDMs were incubated with various protease inhibitors (TAPI-2 [50 μM], Leupeptin [250 μM], BACE IV inhibitor [20 μM], or GM6001 [50 μM]) for 16 hours, and the lysates were analyzed using Western blot to determine CD68 molecular weight changes.

Another possibility is that CD68 protein localized on the plasma membrane could be processed by extracellular proteases under PGRN-deficient conditions, resulting in the changes in MW. To test this, we treated control and *Grn*^*−/−*^ cells with various matrix metalloprotease inhibitors (GM6001 and TAPI-2), β-secretase (BACE1) inhibitor, or leupeptin. None of the inhibitors restored CD68 MW in *Grn*^*−/−*^ cells (Fig. S4), indicating that the decrease in CD68 MW is not likely to be caused by proteolytic processing of the protein.

**Figure 6:**
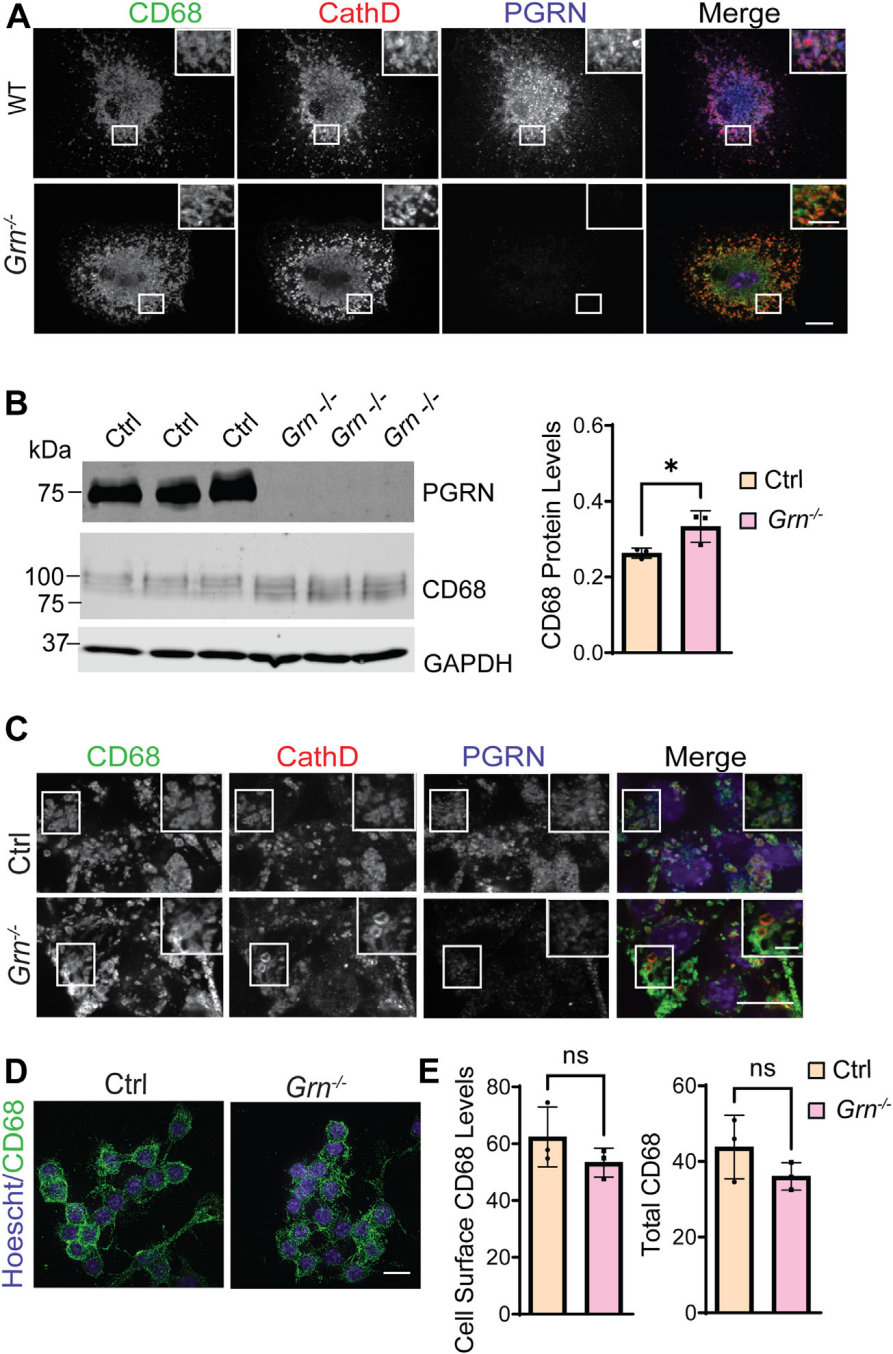
**PGRN does not affect CD68 localization.***A*, WT and *Grn*^*−/−*^ BMDMs were stained with antibodies against CD68, cathepsin D, and PGRN. The scale bar represents 10 μm (*inset*: 5 μm). *B*, deletion of PGRN in RAW264.7 cells leads to the decreased molecular weight of CD68 and increased CD68 levels. CD68 levels were quantified and normalized to GAPDH. n = 3, ∗*p* < 0.05, unpaired *t-*test. *C*, control and *Grn*^*−/−*^ RAW264.7 cells were fixed, permeabilized, and stained with anti-CD68, cathepsin D, and PGRN antibodies. The scale bar represents 10 μm (*inset*: 2.5 μm). *D*, live control and *Grn*^*−/−*^ RAW264.7 cells were incubated with rat anti-mouse CD68 antibodies on ice followed by washing, fixation, blocking, and staining with secondary antibody and Hoechst. Representative images from three replicates are shown. The scale bar represents 10 μm. *E*, cell surface and total levels of CD68 were quantified by ImageJ for experiments in *C* and *D*. n = 3. BMDM, bone marrow–derived macrophage; ns, not significant; PGRN, progranulin.

Next, we examined whether CD68 localization is altered by PGRN loss. In *Grn*^*−/−*^ BMDMs and RAW264.7 cells, CD68 still localizes to the lysosome as indicated by the colocalization between CD68 and the lysosomal marker, cathepsin D (Fig. 6, *A*–*C*). Since CD68 is also present in the plasma membrane, we investigated whether the levels of CD68 at the cell surface are altered in *Grn*^*−/−*^ cells. To test this, we measured cell surface levels of CD68 in nonpermeabilized conditions in control and *Grn*^*−/−*^ RAW264.7 cells (Fig. 6*D*) and found that CD68 surface levels were not altered in *Grn*^*−/−*^ RAW264.7 cells (Fig. 6, *D* and *E*).

## Discussion

### Differential regulation of PGRN-derived granulin peptides

PGRN is processed into individual granulin peptides in the lysosome, and these granulin peptides are proposed to possess unique functions to regulate lysosomal activities (15, 34). Although the granulin peptides are derived from the same precursor, our recent studies have shown that the levels of individual granulin peptides differ from each other (28). This is further supported by our observation that CD68 ablation specifically decreases the levels of granulin E but no other granulins tested (Fig. 4, *A* and *B*). How CD68 regulates the levels of granulin E remains to be determined. Since we failed to detect granulins in the extracellular space, it is unlikely that CD68 regulates the levels of granulin E *via* modulating its secretion or endocytosis, although CD68 can mediate the uptake of granulin E to the lysosome when overexpressed in COS-7 cells (Fig. S1). Instead, we hypothesize that CD68 is required for the stability of granulin E in the lysosomal compartment. It is possible that each granulin peptide interacts with a different set of lysosomal proteins, which determine the stability and half-life of these peptides in the lysosome. Since CD68 is not expressed in neurons, there might exist another binding partner for granulin E in neurons and other cell types to stabilize granulin E.

Since CD68 is critical for maintaining proper levels of granulin E, we expect that granulin E-dependent lysosomal activities should be altered in CD68-deficient cells. Unfortunately, we still do not have a full understanding of granulin E functions in the lysosome. In the literature, GCase (31) and cathepsin D (19, 21) have been shown to be regulated by granulin E, but their activities are not altered by CD68 ablation (Fig. S2, *A* and *B*). Future work is needed to fully dissect the functions of granulin E in the lysosome and the consequence of CD68 ablation on granulin E-dependent lysosomal activities.

### Regulation of CD68 by PGRN and granulin E

Our data have shown that PGRN deficiency results in a shift in the MW of CD68 in addition to an increase in CD68 levels (Figs. 5, *A*–*C* and 6*B*). The MW shift is not caused by alterations in N-glycosylation or proteolytic cleavage. It is possible that loss of PGRN leads to alterations in the O-glycosylation pattern of CD68, since mouse CD68 contains 26 O-linked glycosylation sites (29). Another important question is whether the function of CD68 is affected by PGRN loss. Unfortunately, although CD68 is frequently utilized as a marker for macrophages and activated microglia, little is known about its molecular functions (29). Interactions between CD68 and phosphatidylserine and oxidized low-density lipoprotein on the plasma membrane have been reported, but the physiological importance of this interaction remains to be confirmed (29, 35, 36). A better understanding of CD68 functions will help determine the significance of the observed shift in MW under PGRN-deficient conditions. Nevertheless, our work has identified CD68 as a specific binding partner for granulin E and indicated that regulation of CD68 might be one function of granulin E in the lysosome.

## Experimental procedures

### Primary antibodies and reagents

The following antibodies were used in this study: rat antimouse CD68 (Bio-Rad; catalog no.: MCA1957), rabbit anti-CD68 (Abcam; catalog no.: 125212), sheep antimouse PGRN (R&D Systems; catalog no.: AF2557), mouse anti-Myc (catalog no.: 9E10; Hybridoma Bank), mouse anti-GAPDH (Proteintech Group; catalog no.: 60004-1-Ig), rat antimouse LAMP1 (BD Biosciences; catalog no.: 553792), goat anti-CathD (R&D Systems; catalog no.: AF1029) and rabbit anti-IBA-1 (Wako; catalog no.: 01919741), rabbit anti-PGAM1 (Proteintech; catalog no.: 16126-1-AP), and GFP-Trap were from ChromoTek. Rabbit anti-GFP antibodies were a gift from Professor Anthony Bretscher and rabbit anti-cathepsin D antibodies were a gift from Dr. William Brown at Cornell University. Antibodies against each granulin peptide have been previously characterized (28). Rabbit anti-mouse PSAPs were generated by Pocono Rabbit Farm and Laboratory and previously characterized (14). Alexa Fluor–conjugated secondary antibodies (488/594/647 nm) were from Invitrogen. IRDye 680RD/800CW secondary antibodies were from Invitrogen and LI-COR Biosciences.

The following reagents were also used in the study: Dulbecco’s modified Eagle’s medium (DMEM) (Cellgro; catalog no.: 10-017-CV), 0.25% trypsin (Corning; catalog no.: 25-053-CI), Odyssey blocking buffer (LI-COR Biosciences; catalog no.: 927-40000), protease inhibitor (Roche; catalog no.: 05056489001), Hochest 33342 (Invitrogen), bafilomycin (Sigma; catalog no.: 11707), chloroquine (Sigma; catalog no.: C6628), PNGaseF (New England Biolabs; catalog no.: P0704S), Pierce Bicinchoninic Acid (BCA) Protein Assay Kit (Thermo Fisher Scientific; catalog no.: 23225), OCT compound (Electron Microscopy Sciences, catalog no.: 62550-01), leupeptin (Sigma; catalog no.: L2884), TAPI-2 (EMD Millipore; catalog no.: 579052), GM6001 (EMD Millipore; catalog no.: CC1010), and β-secretase (BACE) IV inhibitor (EMD Millipore; catalog no.: 565788).

### Plasmids

Human CD68 was cloned in the pEGFPs-N2, pcDNA3.1/myc-His A, and the lentiviral vector pCDH-CMV-MCS-EF1-Puro vector (System Biosciences). The pAP5 (Gen Hunter; catalog no.: Q202) vector was used to clone AP-tagged PGRN and granulins A–G. PGRNΔE (amino acids 17–492) was cloned into the pSectag2A vector with an N-terminal FLAG tag.

About 2815 cDNAs encoding the TM proteins from the human ORFeome 8.1 library in the pDONR223 vector were obtained from DNASU and cloned into the pDEST47 vector (Invitrogen) *via* gateway cloning.

### Mouse studies

WT C57/BL6, *Cd68*^*−/−*^ (37), and *Grn*^*−/−*^ (38) mice were purchased from Jax Laboratories. All animals (one to six adult mice per cage) were housed in a 12 h light/dark cycle. Mixed males and females are used in the study. All animal procedures have been approved by the Institutional Animal Care and Use Committee at Cornell University (2017-0056). For perfusion, mice were anesthetized with isoflurane at a concentration of 1 ml isoflurane for every 500 ml of the volume of the anesthesia chamber. The mice were monitored closely, and deep anesthesia was confirmed by toe pinching (1–2 min after induction). Mice were perfused with PBS afterward. To culture primary microglia, P0–P2 pups were decapitated using a razor blade and brains were harvested.

### Cell culture

Human embryonic kidney 293T and RAW264.7 cells were maintained in DMEM (Cellgro) supplemented with 10% fetal bovine serum (Sigma) in a humidified incubator at 37 °C with 5% CO_2_. RAW264.7 cells with PGRN deletion or controls were generated by infecting the cells with lentivirus-expressing Cas9 and the PGRN-targeting guide RNAs (5′-caccgCGGACCCCGACGCAGGTAGG-3′ and 5′-aaacCCTACCTGCGTCGGGGTCCGc-3′) or Cas9 only. Cells were selected with puromycin (8 μg/ml) 2 days after infection, and the knockout is confirmed by Western blot and immunostaining. For transient overexpression, cells were transfected with polyethyleneimine as previously described (39). BMDMs from WT and *Grn*^*−/−*^ mice were cultured according to published protocols (40). Briefly, the cells were flushed out from the femur, tibia, and fibula of mice using DMEM. Cells were spun, filtered, and resuspended in L929 media with 10% fetal bovine serum, and DMEM. Cells were counted with a hemocytometer (Lumictyte; catalog no.: 090001) and plated. Mouse primary cortical neurons and microglia were cultured from P0 to P2 pups according to published protocols (32, 41).

### AP-based cell surface binding screen

COS-7 cells grown in 96-well tissue culture plates (Corning) were transfected with 50 to 100 ng of expression construct for an individual TM protein in each well. Sortilin was used as a positive control for PGRN binding, and pEGFP-C1 vector was used as a negative control. After 2 days, the wells were washed 2× with HBH (Hank’s balanced salt solution with calcium and magnesium with 20 mM Hepes, pH 6.4, and 0.1% bovine serum albumin) and incubated with conditioned medium containing AP-PGRN, AP-PGRN + AP-PSAP, or a mixture of AP-granulin A, B, D, E, and G at 50 nM. AP fusion protein conditioned media were diluted to working concentrations in HBH. About 100 μl was added to each well, and the plate was incubated for 3 h at room temperature. Following the incubation, wells were washed twice with cold HBH to remove traces of unbound AP-fused ligand, and then the cells were fixed with 3.7% formaldehyde at room temperature for 20 min. Wells were washed 1× with HBH, and then GFP expression was assessed by fluorescence microscopy using the ImageXpress system (Molecular Devices). Wells were then filled with HBH, and the plate was sealed tightly with adhesive film (VWR). The plate was incubated overnight at 65 °C to inactivate endogenous APs. Wells were washed 1× with AP buffer (100 mM Tris–HCl, pH 9.5, 100 mM NaCl, and 5 mM MgCl_2_) and incubated with 100 μl of developing solution composed of AP buffer with 350 μg/ml nitroblue tetrazolium chloride (NBT) and 175 μg/ml 5-bromo-4-chloro-3′-indolyl phosphate p-toluidine salt at room temperature for a minimum of 30 min or until signal developed. Positive interactions, as indicated by the presence of an insoluble black-purple precipitate, were confirmed by eye using brightfield microscopy.

### Immunoprecipitation and Western blot analysis

Cells were lysed in a cold solution containing 150 mM NaCl, 50 mM Tris–HCl (pH 7.5), 1% Triton X-100, 0.1% deoxycholic acid, 1× protease inhibitors (Roche). After centrifugation at 14,000*g*, for 15 min, at 4 °C, supernatants were transferred to clean tubes on ice, to which GFP-Trap or Myc-Trap beads (ChromoTek) were added and then rocked for 3 to 4 h at 4 °C. Samples were run on 12% polyacrylamide gels and transferred to Immobilon-FL polyvinylidene fluoride membranes (Millipore Corporation). Membranes were blocked with either 5% nonfat milk in 1× PBS or Odyssey Blocking Buffer (LI-COR Biosciences) for 1 h at room temperature followed by incubation with primary antibodies and left rocking overnight at 4 °C. Membranes were then washed with Tris-buffered saline with 0.1% Tween-20 for three times, 5 min each, and incubated with fluorescently tagged secondary antibodies (LI-COR Biosciences) for 1 h at room temperature, and then followed by three washes. Membranes were scanned using an Odyssey Infrared Imaging System (LI-COR Biosciences). Densitometry was performed using Image Studio (LI-COR Biosciences) and ImageJ (National Institutes of Health).

For granulin peptide detection, NuPAGE Bis–Tris gradient gels (Invitrogen; NP032C and NP032A) were used. Gels were run in NuPAGE Mes SDS cold running buffer (50 mM Mes, 50 mM Tris base, 0.1% SDS, 1 mM EDTA, pH 7.3) and transferred using cold NuPAGE Transfer buffer (25 mM bicine, 25 mM Bis–Tris [free base], 1 mM EDTA, pH 7.2, 20% methanol—final volume) onto a nitrocellulose membrane with 0.2 μm pore size (Millipore Corporation). Membranes were blocked using 5% nonfat milk in 1× PBS for 2 h at room temperature followed by incubation with primary antibody for 2 days. Washes and secondary antibody incubation were performed as aforementioned.

To prepare tissue lysates, mice were perfused with PBS, and tissues were dissected and snap-frozen with liquid nitrogen and kept at −80 °C. On the day of the experiment, frozen tissues were thawed and homogenized on ice with a bead homogenizer (Moni International) in a cold solution of radioimmunoprecipitation assay buffer (150 mM NaCl, 50 mM Tris–HCl [pH 8.0], 1% Triton X-100, 0.5% sodium deoxycholate, and 0.1% SDS) with proteinase inhibitors. After centrifugation at 14,000*g* for 15 min at 4 °C, supernatants were collected. Protein concentrations were determined *via* BCA assay and then standardized. Equal amounts of protein were analyzed by Western blotting using the indicated antibodies.

### GCase activity assay

Spleen tissues from 10-month-old WT and *Cd68*^*−/−*^ mice were homogenized with 0.2% (w/v) sodium taurocholate and 0.1% (v/v) Triton X-100. Protein concentrations were determined *via* BCA assay and then standardized. Tissue lysates were then incubated with 100 nM MDW941 at 37 °C for 30 min. Reactions were stopped by the addition of an equal volume of 2× Laemmli sample buffer with 10% β-mercaptoethanol before heating at 95 °C for 5 min. An equal amount of each sample (50 μg total protein) was run on a 12% polyacrylamide gel, which was scanned at 532 nm excitation/580 nm emission with a Typhoon Imaging System (GE Healthcare), then Western blot and assessment were performed as described previously, with all values normalized to GAPDH.

### Cathepsin D activity assay

Spleen tissues from 6.5-month-old WT and *Cd68*^*−/−*^ mice were lysed in lysis buffer (0.2% [w/v] taurocholate and 0.2% [v/v] Triton X-100 at pH 5.2 at a 1:10 ratio of tissue weight (g) to lysis buffer (ml). Equal amounts of tissue lysates were incubated at 37 °C for 30 min in 100 μl assay buffer (50 mM sodium acetate [pH 5.5], 0.1 M NaCl, 1 mM EDTA, and 0.2% [v/v] Triton X-100) in the presence of the fluorogenic substrate (MOCAc-GKPILF∼FRLK(Dnp)-D-R-NH2) (Calbiochem; catalog no.: 219360). The fluorescence released as a result of cathepsin D proteolytic activity was read at 340 nm (excitation) and 420 nm (emission).

### ELISA

Blood samples were collected from four groups of 3- to 4-month-old mice and left at 4 °C overnight. Blood was spun at 3500*g* for 15 min two times, and serum was collected and analyzed using mouse PGRN ELISA Kit (R&D Systems; catalog no.: MPGRN0).

### Immunofluorescence staining, image acquisition, and analysis

Cells were fixed, permeabilized with 0.05% saponin, and visualized using immunofluorescence microscopy as previously described (42). For cell surface staining, live cells were incubated on ice for 2 h with antibodies recognizing extracellular domain of LAMP1 or CD68 in 1× Hank’s balanced salt solution with 20 mM Hepes, followed by fixation in 4% paraformaldehyde for 20 min at room temperature, then blocked with Odyssey blocking buffer, and followed with secondary antibody incubation with Hoechst for 1 h. The intensity of cell surface LAMP1 and the ratio between cell surface CD68 and total CD68 was quantified using ImageJ from three independent experiments (50–70 cells/experiment). The quantitative analysis of fluorescence images was performed using ImageJ. For the quantitative analysis of intracellular levels of LAMP1, the entire cell body was selected, and the fluorescence intensity was measured directly using ImageJ after a threshold application.

Mice were perfused and fixed with 4% paraformaldehyde in 1× PBS for 2 days, followed by daily incubations with 15% sucrose and then 30% sucrose in 1× PBS, respectively. About 18 μm brain sections were cut with a cryotome. The brain sections were blocked and permeabilized with 0.1% saponin in Odyssey buffer (Licor) followed by incubation with primary and secondary antibodies as mentioned previously.

### Deglycosylation assay

Spleens were harvested from WT, *Cd68*^*−/−*^, and *Grn*^*−/−*^ mice and lysed in cold IP lysis buffer (150 mM NaCl, 50 mM Tris [pH 8.0], 1% Triton X-100, 0.1% deoxycholic acid, and 1× protease inhibitors). The samples were centrifuged at 14,000*g* for 15 min at 4 °C, and the supernatants were collected. After centrifugation, protein concentrations were quantified using the Pierce Protein Assay Kit (catalog no.: 23225). The samples were then incubated with 10 μl of protein G beads (Genscript; catalog no.: L00209) and placed in a nutator for 1 h at 4 °C. The samples were then centrifuged at 3000*g* for 30 s at 4 °C, and the supernatant was collected and placed in clean tubes, to which antibodies against CD68 were added and rocked for 3 to 4 h. About 15 μl of protein G-beads were then added and rocked for 2 h at 4 °C. Samples were centrifuged at 3000*g* for 30 s at 4 °C and then washed with 1 ml of a solution containing 150 mM NaCl, 50 mM Tris (pH 8.0), and 1% Triton X-100. This was repeated for a total of three washes. After the final centrifugation, 200 μl of IP wash buffer was added and divided into two clean tubes and centrifuged. One tube was eluted by the addition of 25 μl of Laemmli sample buffer with 5% β-mercaptoethanol, whereas the other tube was used to perform the deglycosylation assay (NEB; catalog no.: P0704S).

For the deglycosylation assay, 1 μl of glycoprotein denaturing buffer (10×) and 9 μl H_2_O were added to the tube. The sample was heated to 100 °C for 10 min and then placed on ice for 10 s 2 μl of GlycoBuffer 2 (10×), 2 μl of 10% NP-40 and 6 μl of water was added, and the sample was lightly flicked. Finally, 1 μl of PNGase F was added, mixed gently, and incubated at 37 °C for 1 h. Western blot was performed of the input, IP, and deglycosylated samples.

### Statistical analysis

All statistical analyses were performed using GraphPad Prism 8 (GraphPad Software, Inc). All data are presented as mean ± SEM. Statistical significance was assessed by unpaired *t-*test (for two-group comparisons). *p* Values less than or equal to 0.05 were considered statistically significant. ∗*p* < 0.05; ∗∗*p* < 0.01; ∗∗∗*p* < 0.001; and ∗∗∗∗*p* < 0.0001.

## Data availability

The data supporting the findings of this study are available from the corresponding author on request.

## Supporting information

This article contains supporting information.

## Ethical approval and consent to participate

All applicable international, national, and/or institutional guidelines for the care and use of animals were followed. The work under animal protocol 2017-0056 is approved by the Institutional Animal Care and Use Committee at Cornell University.

## Conflict of interest

The authors declare that they have no conflicts of interest with the contents of this article.
