# Supplementary material for: Progranulin-derived granulin E and lysosome membrane protein CD68 interact to reciprocally regulate their protein homeostasis
Source: J Biol Chem. 2022 Aug 4;298(9):102348. doi: 10.1016/j.jbc.2022.102348 (PMC9450144; doi:10.1016/j.jbc.2022.102348)
Supplement: Supplementary Material [file mmc1.docx]

**Supplementary Figures**

**Supplementary Figure 1: CD68 can mediate the uptake of PGRN and granulin E when overexpressed.** COS-7 cells were transfected with vector control, CD68 or sortilin expressing constructs and incubated with conditioned medium (CM) containing PGRN or granulin E as indicated. The cells were washed and fixed 4 hours after incubation and stained with CD68 and PGRN antibodies. Scale bar=10 μm.

**Supplementary Figure 2: CD68 deficiency does not affect cathepsin D or GCase activities.**

**A.** Spleen lysates from 6.5-month-old WT and *Cd68^-/-^* mice were incubated with fluorogenic cathepsin D substrate. Fluorescence was read at 340nm. 3 mice per genotype were analyzed (n=3). ns, not significant. **B.** Spleen lysates of 10-months-old WT and *Cd68^-/-^* mice were incubated with the GCase activity probe, MDW941. The samples were run on SDS-PAGE and MDW941 labeled GCase was detected using a fluorescent scanner at 532nm excitation. Western blot was performed using anti-PGAM1 antibodies as a loading control. 3 mice per genotype were analyzed (n=3). ns, not significant.

**Supplementary Figure 3: CD68 deficiency does not affect Prosaposin processing.**

**A.** Spleen lysates from 10-month-old WT and *Cd68^-/-^* mice were analyzed via western blot and probed with anti prosaposin (PSAP) antibodies. The levels of PSAP and saposin peptides were quantified and normalized to GAPDH. 5 mice per genotype were analyzed (n=5). ns, not significant; unpaired t-test.

**Supplementary Figure 4: Inhibition of proteases does not rescue CD68 molecular weight changes in PGRN deficient cells.**

WT and *Grn^-/-^* BMDMs were incubated with various protease inhibitors (TAPI-2 (50μM), Leupeptin (250μM), BACE IV inhibitor (20μM), or GM6001(50μM)) for 16 hours and the lysates were analyzed using western blot to determine CD68 molecular weight changes.
